# The clinicopathological and prognostic significances of *CDC73* expression in cancers: a bioinformatics analysis

**DOI:** 10.18632/oncotarget.20446

**Published:** 2017-08-24

**Authors:** Hua-Chuan Zheng, Bao-Cheng Gong, Shuang Zhao

**Affiliations:** ^1^ Department of Experimental Oncology and Animal Center, Shengjing Hospital of China Medical University, Shenyang 110004, China

**Keywords:** CDC73, bioinformatics analysis, cancers

## Abstract

CDC73 interacts with human PAF1 complex, histone methyltransferase complex and RNA polymerase II for transcription elongation and 3’ end processing. Its down-regulated expression was immunohistochemically detected in gastric, colorectal, ovarian and head and neck cancers, and positively correlated with aggressive behaviors and unfavorable prognosis of malignancies. We performed a bioinformatics analysis by using Oncomine, TCGA and KM plotter databases. It was found that *CDC73* mRNA was overexpressed in gastric, lung, breast and ovarian cancers, even stratified by histological subtypes (p<0.05). *CDC73* mRNA expression was stronger in gastric intestinal- than diffuse-type carcinomas (p<0.05), and positively correlated with distant metastasis and TNM staging of lung cancer (p<0.05). *CDC73* mRNA expression was positively related to both overall and progression-free survival rates of the patients with gastric cancer, even stratified by gender, lymph node involvement, or treatment (p<0.05), while versa for breast cancer (p<0.05). The prognostic significance of *CDC73* mRNA was dependent on the datasets and pathological grouping in lung and ovarian cancers. These findings indicated the *CDC73* mRNA overexpression was positively linked to carcinogenesis. It is cautious to employ *CDC73* mRNA to evaluate the clinicopathological behaviors and prognosis of cancers.

## INTRODUCTION

The hyperparathyroidism-jaw tumor (HPT-JT) syndrome is an autosomal dominant disorder characterized by occurrence of parathyroid tumors, atypical adenomas and carcinomas, ossifying jaw fibromas, renal tumors and uterine benign and malignant neoplasms, and caused by *HRPT2* (also called *CDC73*) mutation. It is located on human chromosome 1q31.2 and encodes a 531-aa parafibromin [1]. In nucleus, parafibromin interacts with human PAF1 complex (including PAF1, LEO1, and CTR9), and RNA polymerase II for transcription elongation and 3’ end processing [2, 3]. It can also bind to a histone methyltransferase complex that methylates histone H3 on lysine 4 [4]. Parafibromin methylates histone H3K9 to repress Cyclin D1 expression via the interaction with the histone methyltransferase, SUV39H1 [5]. Reportedly, the interaction between parafibromin and the ring finger proteins RNF20/40 was essential for the histone 2B monoubiquitination [6]. In cytosol, parafibromin physically binds to eEF1Bγ and hSki8 for destabilizing p53 mRNA and suppressing p53-mediated apoptosis [7]. Wei et al. [8] found that parafibromin interacted with JAK1/2, promoted the interactions of JAK1- JAK2 and JAK1/2-STAT1, and enhanced tyrosine phosphorylation of STAT1 by JAKs after IFN-γ stimulation. Kikuchi et al. [9] demonstrated that dephosphorylated parafibromin, mediated by SHP2 phosphatase and attenuated by PTK6, competitively interacted with β-catenin and Gli1, thereby potentiating transactivation of Wnt- and Hh-target genes in a mutually exclusive manner. Consequently, acute loss of parafibromin in mice disorganizes the normal epithelial architecture of the intestine, which requires coordinated activation/inactivation of Wnt, Hh and/or Notch signaling. Agarwal et al. [10] demonstrated that parafibromin bound to muscle alpha- actinins (actinin-2 and actinin-3).

Wang et al. [11] reported that *HRPT2* deletion was lethal at embryonic day 6.5 (E6.5). Controlled deletion of *HRPT2* after E8.5 resulted in apoptosis and growth retardation. *HRPT2* deletion in adult mice led to severe cachexia and death within 20 days. Walls et al. [12] found that mice with *HRPT2* deletion developed parathyroid and uterine tumors, and might be considered as a model for hyperparathyroidism-jaw tumor syndrome. Previously, we investigated the clinicopathological and prognostic significances of parafibromin expression in gastric, colorectal, ovarian, lung, head and neck cancers, and found its down-regulated expression and its inverse link with aggressive behaviors and unfavorable prognosis [13–18]. Parafibromin expression was found to negatively correlate with tumor size, pathological stage, lymphovascular invasion and C-erbB2 expression of breast cancer [19, 20]. There was an inverse correlation between lymph node metastasis or depth of invasion and parafibromin expression in urothelial carcinoma [21]. The down- regulation or loss of parafibromin expression could be also employed as a novel marker of tumor progression or aggressiveness in laryngeal squamous cell carcinoma [22]. In the present study, we aimed to explore the clinicopathological roles of *CDC73* mRNA expression in various cancers using bioinformatics analysis, including gastric, lung, breast and ovarian cancers.

## RESULTS

### The clinicopathological significances of *CDC73* expression in gastric cancer

**Figure 1:**
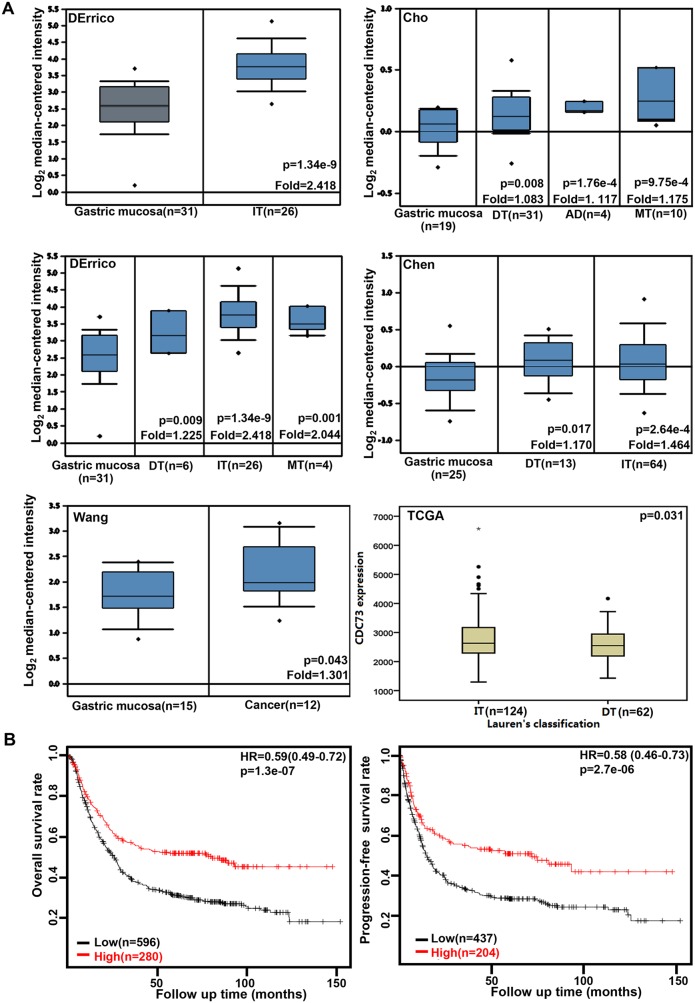
The clinicopathological significances of *CDC73* expression in gastric cancer DErrico’s, Cho’s, Chen’s, and Wang's datasets were used for bioinformatics analysis to explore *CDC73* mRNA expression in gastric cancer. A higher *CDC73* expression was detectable in gastric cancer than that in normal mucosa, even stratified into intestinal- (IT), diffuse- (DT), and mixed-type (MT) carcinomas by Lauren's classification (**A**, p<0.05). TCGA database shows that *CDC73* mRNA was more expressed in IT than DT ones (A, p<0.05). According to the data from KM plotter, *CDC73* mRNA expression was positively related to both overall and progression-free survival rates of the patients with gastric cancer (**B**, p<0.05). AD, adenocarcinoma; HR, hazard ratio.

We collected the results from DErrico’s, Cho’s, Chen’s, Wang’s, and TCGA's datasets and analyzed *CDC73* mRNA expression in gastric cancer. A higher *CDC73* expression was found in gastric normal mucosa than that in cancer, even stratified into intestinal-, diffuse-, and mixed-type carcinomas by Lauren's classification (Figure 1A, p<0.05). *CDC73* mRNA was more expressed in intestinal- than diffuse-type carcinomas (Figure 1A, p<0.05). As shown in Figure 1 and Table 1, *CDC73* mRNA expression was positively related to both overall and progression-free survival rates of the patients with gastric cancer, even stratified by gender, lymph node involvement, or treatment (p<0.05). It was the same for overall survival rate in the patients with gastric cancer, stratified by distant metastasis, or Lauren's classification (p<0.05). A higher *CDC73* mRNA expression was positively correlated with overall and progression-free survival rates of Her2-netagive cancer patients (p<0.05). Stage-I, stage-III, T_2_ and T_3_ cancer patients with high *CDC73* mRNA expression showed a long overall survival time than those with its low expression (p<0.05), while it was the same for progression-free survival in the patients with stage-III and -IV, T_2_, M0, and diffuse-type carcinoma (p<0.05).

**Table 1:** The prognostic significances of *CDC73* mRNA in gastric cancer

Clinicopathological features	Overall survival		Progression-free survival	
	Hazard ratio	p	Hazard ratio	p
Sex				
Female	0.43 (0.28 − 0.65)	5.7e−5	0.43 (0.28 − 0.67)	0.00011
Male	0.54 (0.43 − 0.68)	9.8e−8	0.63 (0.49 − 0.81)	0.00036
TNM staging				
I	0.28 (0.1 − 0.76)	0.0078		
III	0.59 (0.43 − 0.81)	0.00084	0.62 (0.39 − 0.99)	0.042
IV			0.64 (0.42 − 0.98)	0.041
T				
2	0.55 (0.36 − 0.84)	0.0052	0.61 (0.39 − 0.94)	0.022
3	0.71 (0.5 − 1)	0.049		
N				
0	0.28 (0.12 − 0.67)	0.0021	0.33 (0.14 − 0.76)	0.0064
1-3	0.62 (0.47 − 0.82)	0.00065	0.66 (0.5 − 0.88)	0.004
1	0.52 (0.34 − 0.81)	0.0034	0.63 (0.41 − 0.95)	0.025
2	0.5 (0.3 − 0.84)	0.0073	0.4 (0.22 − 0.72)	0.0018
M				
0	0.57 (0.43 − 0.76)	0.00011	0.61 (0.45 − 0.82)	0.00087
1	0.45 (0.23 − 0.86)	0.014		
Treatment				
Surgery alone	0.6 (0.44 − 0.83)	0.0014	0.66 (0.49 − 0.91)	0.011
Other adjuvant	0.42 (0.17 − 1.03)	0.05	0.41 (0.18 − 0.91)	0.024
Lauren's classification				
Intestinal-type	0.55 (0.4 − 0.75)	0.00017		
Diffuse-type	0.56 (0.4 − 0.79)	8e−4	0.55 (0.36 − 0.84)	0.0047
Mixed-type	0.35 (0.12 − 1.03)	0.046		
Her2 positivity				
-	0.49 (0.38 − 0.64)	3.3e−8	0.49 (0.36 − 0.66)	2.5e−6

### The clinicopathological significances of *CDC73* expression in lung cancer

**Figure 2:**
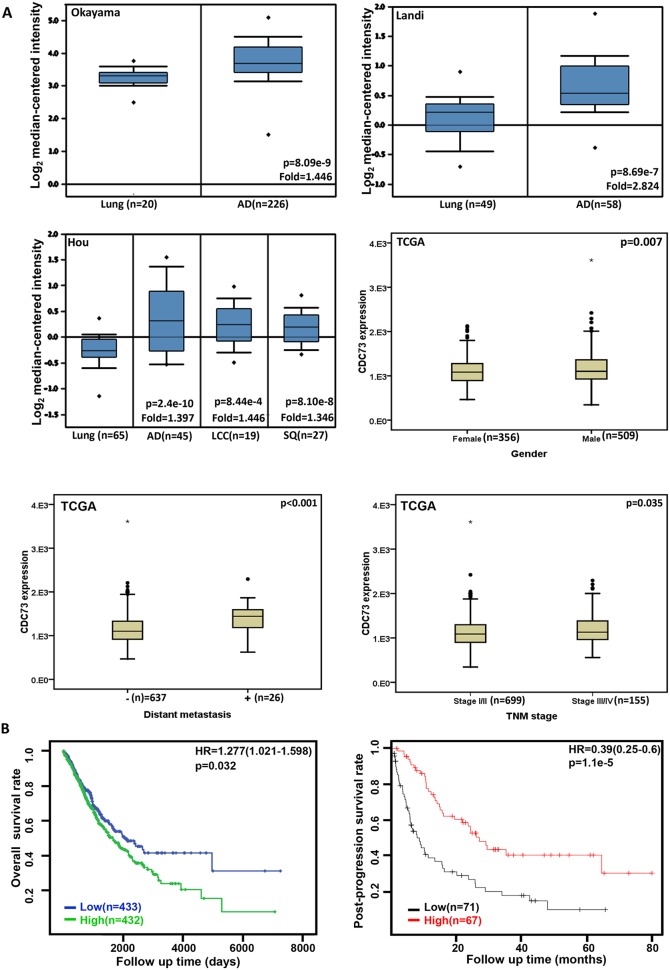
The clinicopathological significances of *CDC73* expression in lung cancer Okayama, Landi’s, and Hou's datasets were employed for bioinformatics analysis to determine *CDC73* mRNA expression in lung cancer. A higher *CDC73* mRNA expression was detectable in lung adenocarcinoma (AD), large cell carcinoma (LCC) and squamous cell carcinoma (SQ) than that in normal lung tissues (**A**, p<0.05). TCGA database shows that *CDC73* was more expressed in male than female cancer patients (A, p<0.05). *CDC73* mRNA expression was positively correlated with distant metastasis and TNM staging of lung cancer (A, p<0.05). There was negative association between *CDC73* mRNA expression and favorable prognosis of the patients with lung cancer according to TCGA dataset (p<0.05), while versa for KM plotter (**B**, p<0.05). HR, hazard ratio.

We found that a higher *CDC73* expression in lung adenocarcinoma, large cell carcinoma and squamous cell carcinoma than that in normal lung tissues (Figure 2A, p<0.05). *CDC73* mRNA was more expressed in male than female patients with lung cancer (Figure 2A, p<0.05). According to TCGA database, *CDC73* mRNA expression was positively correlated with distant metastasis, TNM staging, and unfavorable prognosis of lung cancer (Figures 2A and 2B, p<0.05). The similar data were obtained for overall survival rate in the patients with Grade-2, M0, N2, T1, or T2 cancer, progression-free survival rate in the patients with squamous, T1 or N1 cancer, and post-progression survival rate in the patients with no smoking or T1 stage according to KM plotter, (p<0.05, data not shown). In contrast, there was positive association between *CDC73* mRNA expression and post-progression survival rate of the patients with lung cancer according to KM plotter (Figure 2B, p<0.05). It was the same for overall survival rate in the patients with adenocarcinoma, margin-negative cancer, stage-I or -II cancer (p<0.05, data not shown), and for progression-free survival rate in the patients with margin-negative cancer, or no smoking (p<0.05, data not shown).

### The clinicopathological significances of *CDC73* expression in breast cancer

**Figure 3:**
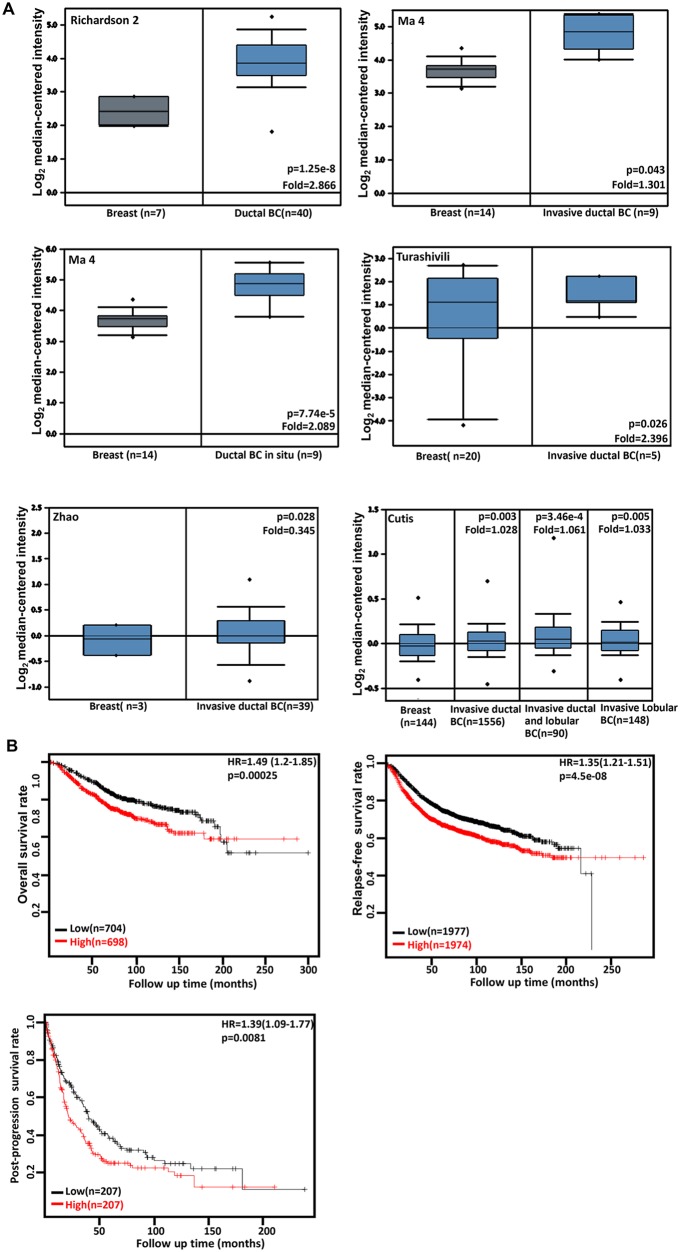
The clinicopathological significances of *CDC73* expression in breast cancer Richardson 2’s, Ma 4’s, Turashivili’s, Zhao's and Cutis's datasets were used for bioinformatics analysis to confirm *CDC73* mRNA expression in breast cancer. A lower *CDC73* mRNA expression was detectable in normal breast than that in ductal and/or lobular breast carcinoma (BC, **A**, p<0.05). According to the data from KM plotter, *CDC73* mRNA expression was positively related to the low overall, relapse-free and post-progression survival rates of the patients with breast cancer (**B**, p<0.05). HR, hazard ratio.

A higher *CDC73* expression was seen in breast ductal and/or lobular carcinoma than normal tissue (Figure 3A, p<0.05). According to the data from KM plotter, *CDC73* mRNA expression was negatively linked to the high overall, relapse-free and post-progression survival rates of the patients with breast cancer (Figure 3B, p<0.05). There was a negative association of *CDC73* expression with the overall, relapse-free, and distant-metastasis-free survival rates of the cancer patients with no lymph node metastasis (p<0.05, data not shown). An inverse association between relapse-free survival rate and *CDC73* expression was seen in the breast cancer patients with ER positive or negative, and wild-type p53, and Luminal-A or -B (p<0.05, data not shown). It was the same for overall survival rate in the patients with ER positive, or Grade 3 cancer (p<0.05, data not shown). *CDC73* expression was negatively correlated with post-progression survival rate of Luminal-A breast cancer patients, or distant-metastasis-free survival rate of PR-positive breast cancer patients respectively (p<0.05, data not shown).

### The clinicopathological significances of *CDC73* expression in ovarian cancer

**Figure 4:**
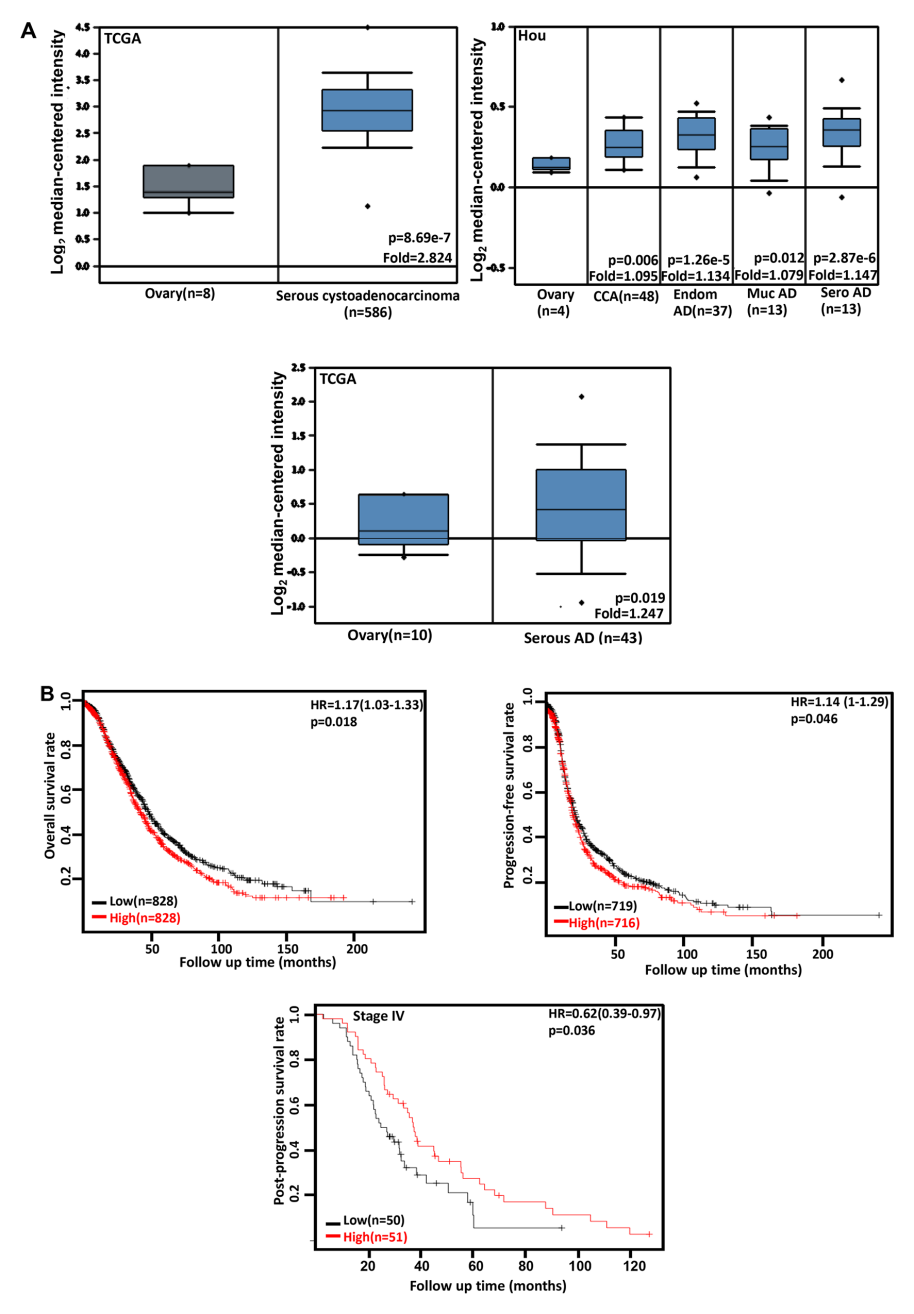
The clinicopathological significances of *CDC73* expression in ovarian cancer TCGA's and Hou's datasets were employed for bioinformatics analysis to observe *CDC73* mRNA expression in ovarian cancer. A lower *CDC73* mRNA expression was detectable in ovary than that in serous cystoadenocarcinoma, clear cell adenocarcinoma (CCA), endometriod (Endom), mucinous (Muc) and serous (Sero) adenocarcinoma (AD, **A**, p<0.05). According to the data from KM plotter, *CDC73* mRNA expression was inversely related to both overall and progression-free survival rates of the patients with ovarian cancer, but versa for post-progression survival rate of the patients with stage-IV ovarian cancer (**B**, p<0.05). Interaction networks of the DEGs. HR, hazard ratio.

We performed bioinformatics analysis of *CDC73* mRNA expression in ovarian cancer using TCGA's and Hou's datasets. There was a lower *CDC73* expression in normal ovary than that in serous cystoadenocarcinoma, clear cell, endometriod, mucinous and serous adenocarcinomas (Figure 4A, p<0.05). The data from KM plotter showed a negative relationship between *CDC73* mRNA expression and either overall or progression-free survival rates of the patients with ovarian cancer (Figure 4B, p<0.05). However, a positive correlation between *CDC73* expression and post-progression survival rate was observed in the patients with stage-IV ovarian cancer (Figure 4B, p<0.05).

## DISCUSSION

As a tumor suppressor, parafibromin inhibits colony formation and cellular proliferation, and causes G_1_ phase arrest in cervical cancer cells [23]. Wild-type parafibromin is located in the nucleus of osteosarcoma cells, responsible for apoptotic induction and G_1_ phase arrest via MEK/ERK and PI3K/Akt signaling inactivation, and Caspase activation [24]. Parafibromin knockdown results in uncleaved histone mRNA with polyadenylated tails [25], and a high proliferation by both c-myc protein stabilization and activation of the c-myc promoter [26]. In contrast, SHP2 tyrosine phosphatase converts parafibromin from a tumor suppressor to an oncogenic driver by its interaction with and subsequent stablization of β-catenin to upregulate expression of Wnt target genes, including cyclin D1 and c-myc [27]. We speculate that parafibromin plays a role of two-side sword in cancer cells.

Masi et al. [28] characterized a novel somatic *CDC73* missense mutation (Ile60Asn) caused reduced nuclear parafibromin immunoreactivity. Overexpression of Ile60Asn mutant led to increased cell proliferation and to accumulation in the G_2_/M phase of cell cycle with the ability to down-regulate c-myc expression lost. Parafibromin protein was found in the cilia of pseudo-stratified bronchial and fallopian epithelium [16, 17]. In our previous work [29], WT parafibromin overexpression was found to suppressed proliferation, tumor growth, induced cell cycle arrest and apoptosis in colorectal cancer cells, but it was the converse for mutant-type (MT, mutation in nucleus localization sequence) parafibromin. According to transcriptomic analysis, WT parafibromin suppressed PI3K-Akt and FoxO signaling pathways, while MT one promoted PI3K-Akt pathway, focal adhesion, and regulation of actin cytoskeleton. These findings suggested that subcellular distribution of parafibromin determined its biological functions.

Reportedly, parafibromin expression was down-regulated during gastric, colorectal and head and neck carcinogenesis, and inversely linked to such aggressive behaviors as tumor size, depth of invasion, lymphatic invasion, lymph node metastasis or TNM staging by inducing apoptosis and cell cycle arrest, and suppressing proliferation, migration, invasion, and epithelial-mesenchymal transition [13, 15, 18]. At mRNA level, *CDC73* hypoexpression was detectable in colorectal, lung and ovarian cancers by real-time RT-PCR or in situ hybridization [15–17], but *CDC73* mRNA was found to up-regulated in gastric, lung, breast and ovarian cancers by transciptomic sequencing in the present study. Additionally, it was positively correlated with distant metastasis and TNM staging of lung cancer. The paradoxical results might be due to different approaches and tissue specificity. If *CDC73* mRNA overexpression was true, we concluded that its up-regulation might be a feedback reaction in malignancies or acts as an oncogene in some situation.

In line with our results, there was stronger positivity of parafibromin in gastric intestinal- than diffuse-type carcinomas [13], which also was supported by weaker parafibromin expression in signet ring cell carcinoma than the others [14]. However, Shen et al. [17] found parafibromin expression was inversely associated with the differentiation of ovarian cancers. Recently, we reported that *CDC73* expression was higher in moderately- than well-differentiated adenocarcinoma at both mRNA and protein level [29], opposite to our finding. As a differentiation marker, ALP activity was increased in *CDC73* transfectants of colorectal cancer cells [13, 29]. Taken together, we concluded that *CDC73* mRNA underlay the molecular mechanisms of the differentiation of gastric cancer.

Parafibromin expression was found to positively correlate with favorable prognosis of gastric cancer [13] and pulmonary adenocarcinoma [16]. Immunohistochemically, parafibromin expression was employed as an independent factor for a better overall or relapse-free survival of the patients with colorectal cancer [15] or head and neck squamous cell carcinoma [18] respectively. In contrast, the converse was true for cumulative survival rate of the patients with ovarian cancer [17]. In gastric cancer, we found a positive correlation of *CDC73* mRNA expression with both overall and progression-free survival rates, even stratified by gender, lymph node involvement, or treatment. In breast cancer, there was a negative association of *CDC73* expression with the overall, relapse-free, post-progression or distant-metastasis-free survival rates of the patients, even in the subgroups. In TCGA database, *CDC73* mRNA was inversely linked to favorable overall prognosis of lung cancer, which was determined by its positive association with aggressive parameters. However, it was the converse for KM-plotter. As for ovarian cancer, the prognostic significance of *CDC73* expression was dependent on pathological grouping. These results might be attributable to different databases, tissue specificity and distinct grouping. Therefore, it should be careful to employ *CDC73* mRNA as a prognostic marker in clinicopathological practice.

In summary, up-regulated *CDC73* mRNA expression in cancer and its positive correlation with aggressive behaviors and unfavorable prognosis might be due to a feedback reaction or its oncogenic function. The paradoxical results about the prognostic significances of *CDC73* mRNA might also result from different databases, tissue specificity and distinct grouping.

## MATERIALS AND METHODS

### Bioinformatics analysis

The individual gene expression level of *CDC73* was analyzed using Oncomine (www.oncomine.org), a cancer microarray database and web-based data mining platform for a new discovery from genome-wide expression analyses. We compared the differences in *CDC73* mRNA level between normal tissue and cancer. All data were log-transformed, median centered per array, and standard deviation normalized to one per array. The expression (RNA-seqV2) and clinicopathological data of gastric (n=392), lung (n=865), breast (n=1093) and ovarian (n=304) cancer patients were downloaded from Cancer Genome Atlas (TCGA) database by TCGA-assembler in R software. We integrated the raw data and compared *CDC73* expression with clinicopathological and prognostic data of the cancer patients. Additionally, the prognostic significance of *CDC73* mRNA was also analyzed using Kaplan-Meier plotter (http://kmplot.com).

### Statistical analysis

The data from TCGA database was dealt with SPSS 10.0 software using student t test. Kaplan-Meier survival plots were generated with survival curves compared by log-rank statistic. Two-sided p < 0.05 was considered as statistically significant. SPSS 10.0 software was employed to analyze all the data.
